# Polygenic Risk and Linked Metabolic Profile in Systemic Lupus Erythematosus: Cross-Sectional Insights

**DOI:** 10.3390/genes17010053

**Published:** 2026-01-01

**Authors:** Andrea Higuera-Gómez, María Martínez-Urbistondo, Amanda Cuevas-Sierra, Begoña de Cuevillas, Ulises De la Cruz-Mosso, Carolina F. Nicoletti, Jhulia C. N. L. da Mota, Susana Mellor-Pita, Marta Alonso-Bernáldez, Barbara Vizmanos, J. Alfredo Martínez

**Affiliations:** 1 Department of Pharmacy and Nutrition, Faculty of Biomedical and Health Sciences, Universidad Europea de Madrid, Calle Tajo s/n, Villaviciosa de Odón, 28670 Madrid, Spain; 2 Precision Nutrition and Cardiometabolic Health, IMDEA-Food Institute (Madrid Institute for Advanced Studies), Campus of International Excellence (CEI) UAM+CSIC, 28049 Madrid, Spain; 3 Internal Medicine Service, Puerta de Hierro University Hospital of Majadahonda, 28222 Majadahonda, Spain; 4 Red Iberoamericana de Colaboración Académica y Científica en Nutriómicas y Nutrición de Precisión (RINN22), 28049 Madrid, Spain; ulises.mosso@academicos.udg.mx (U.D.l.C.-M.); bvizmanos@yahoo.com.mx (B.V.); 5 Red de Inmunonutrición y Genómica Nutricional en las Enfermedades Autoinmunes, Departamento de Neurociencias, Centro Universitario de Ciencias de la Salud, Universidad de Guadalajara, Guadalajara 44340, Jalisco, Mexico; 6 Laboratorio de Evaluación del Estado Nutricio, Centro de Investigación en Educación y Bienestar Universitario, Centro Universitario de Ciencias de la Salud, Universidad de Guadalajara, Guadalajara 44340, Jalisco, Mexico; 7 Center of Lifestyle Medicine, Faculdade de Medicina FMUSP, Universidade de Sao Paulo, Sao Paulo 01246-903, Brazil; 8 ADNTRO Genetics, Carretera Betlem, s/n, Colonia de Sant Pere, 07579 Arta, Spain; 9 Biomedical Research Centre for Obesity Physiopathology and Nutrition Network (CIBEROBN), Instituto de Salud Carlos III (ISCIII), 28029 Madrid, Spain

**Keywords:** systemic lupus erythematosus (SLE), polygenic risk score (PRS), genetic predisposition, autoimmune diseases, inflammation, cardiovascular risk, precision medicine

## Abstract

**Background/Objectives**: Systemic lupus erythematosus (SLE) is a complex autoimmune disease with a multifactorial origin involving genetic, epigenetic, and environmental determinants as well as some risk factors. Genetic predisposition has been quantified through polygenic risk scores (PRS), which integrate the cumulative effect of multiple single nucleotide variants (SNVs) associated with disease risk. Despite extensive research on immune and inflammatory pathways in SLE, the interplay between genetic susceptibility and metabolic dysfunction remains poorly understood. This study aimed to explore associations between SLE-related PRS and metabolic, inflammatory, and clinical parameters in adults participating in the METAINFLAMACIÓN-CM project (Hospital Universitario Puerta de Hierro Majadahonda, Madrid, Spain). **Methods**: Ninety-three participants were included: 56 SLE patients and 37 individuals with metabolic syndrome (MetS) as a reference group. PRS were computed based on validated lupus-associated SNVs. **Results**: SLE patients showed a distinct metabolic profile compared with the MetS group, characterized by lower BMI, visceral fat, blood pressure, glucose, and liver enzyme levels. Within the SLE cohort, PRS values varied markedly and correlated with specific clinical and biochemical features. Linear regression models revealed a significant inverse association between PRS in SLE and ferritin levels, whereas other metabolic and inflammatory markers (glucose, IL-6, LDL, CRP, neutrophils) were directly influenced by clinical factors. **Conclusions**: Polygenic predisposition contributes to variability in SLE metabolic phenotype but does not independently drive most inflammatory parameters. SLE patients displayed metabolic and inflammatory alterations relevant to cardiovascular risk, highlighting the importance of comprehensive cardiometabolic assessment. Integrating PRS with metabolic profiling may support precision personalized management and improve cardiovascular risk evaluation in SLE.

## 1. Introduction

Systemic lupus erythematosus (SLE) is a multifactorial autoimmune disease characterized by the loss of immunological tolerance to self-antigens and the production of pathogenic autoantibodies, whose etiology involves a complex interplay between genetic, epigenetic, and environmental factors, contributing to heterogeneous clinical phenotypes and disease trajectories [1,2,3].

Genetic susceptibility has long been recognized as a keystone of SLE pathogenesis, with genome-wide association studies (GWAS) identifying more than 300 genetic loci associated with lupus susceptibility across diverse populations, underscoring the disease’s highly polygenic and heterogeneous nature [4]. However, single-variant effects are modest, and the cumulative burden of genetic risk is better captured through polygenic risk scores (PRS), which integrate the weighted influence of multiple alleles into a single quantitative index of inherited predisposition [5]. As described by elsewhere [5], PRS provide valuable tools for quantifying genetic liability in complex diseases and may inform both prediction and mechanistic understanding when combined with clinical and biochemical data. Recent molecular evidence has additionally shown that dysregulated splicing factors and abnormal alternative splicing events contribute to immune dysregulation in SLE, offering further insight into post-transcriptional regulatory mechanisms that complement inherited genetic susceptibility and may represent potential therapeutic targets [6].

In parallel, metabolic disturbances such as dyslipidemia, insulin resistance, and impaired adiposity are frequently observed in SLE patients [7,8,9]. These alterations are often attributed to chronic inflammation, glucocorticoid therapy, and immune activation, yet the interaction between genetic predisposition and metabolic dysregulation remains underexplored. Recent studies have highlighted shared inflammatory pathways between autoimmune and metabolic disorders, reinforcing the concept of immunometabolic cross-talk and suggesting that metabolic dysregulation may amplify or modulate disease expression in SLE [10,11].

Metabolic syndrome (MetS) represents a clinically relevant model of systemic, low-grade inflammation and metabolic impairment in the absence of autoimmunity, providing a useful reference phenotype to disentangle features attributable to metabolic dysfunction from those driven predominantly by autoimmune mechanisms [12].

Therefore, this study aimed to explore associations between SLE-related PRS and metabolic, inflammatory, and clinical parameters. Comparisons with individuals diagnosed with MetS were included to contextualize these parameters against a reference phenotype of systemic inflammation without autoimmunity. This comparative framework provides a clear basis for determining whether the metabolic alterations observed in SLE are linked to genetic susceptibility to the disease or reflect more general inflammatory processes.

## 2. Materials and Methods

### 2.1. Participants

The study included 93 adult participants (aged ≥ 18 years) from the METAINFLAMACIÓN-CM project (reference Y2020/BIO-6600), conducted at the Department of Internal Medicine, Hospital Universitario Puerta de Hierro Majadahonda (Madrid, Spain). Participants comprised 56 individuals diagnosed with SLE (according to EULAR/ACR 2019 criteria) [13] and 37 individuals with MetS following NCEP-ATP III guidelines [14] who were recruited from January 2022 to February 2024. All participants provided written informed consent prior to inclusion.

Patients were selected based on clearly defined inclusion and exclusion criteria. Inclusion criteria for SLE participants were age ≥ 18 years, a confirmed diagnosis of SLE, and a stable disease state defined as SLEDAI < 4, reflecting minimal or no clinical activity. Exclusion criteria included the presence of other autoimmune diseases, active infections, or pregnancy. MetS participants met standard diagnostic criteria, with similar exclusion criteria applied. Screening and eligibility confirmation involved review of clinical records, physical examination, and laboratory assessments to ensure participants met the inclusion and exclusion criteria.

All participants were of predominantly European ancestry. Specifically, among the 56 SLE patients, 49 were Caucasian and 7 were Hispanic, while among the 37 MetS participants, 33 were Caucasian and 4 were Hispanic. This composition is largely consistent with the population used to validate the polygenic risk score (PRS) employed in this study. The small proportion of participants of non-European ancestry is acknowledged as a potential limitation for the generalizability of the PRS.

All SLE patients were under supervised medical treatment prescribed according to European guidelines and departmental clinical experience, minimizing potential biases related to therapeutic interventions.

The study was conducted according to the Declaration of Helsinki and approved by the Ethics Committee of Hospital Universitario Puerta de Hierro Majadahonda (protocol code: File Number PI 164-21).

To ensure sufficient statistical power for genetic association analyses, a sample size calculation was performed using the Fisterra calculator, considering the polygenic risk score (PRS) as the primary quantitative variable. Assuming an expected effect size of 6 units, a standard deviation of 12 units, a significance level of *p* < 0.05, and a desired power of 90%, the estimated minimum sample size was 84 participants, which was exceeded in the current cohort.

### 2.2. Anthropometric, Clinical, and Biochemical Assessment

Anthropometric, clinical, and biochemical parameters were collected under standardized conditions. Anthropometric and body composition variables included heigh, weight, body mass index (BMI), total muscle mass, total fat mass, visceral fat, biological age, waist and hip circumference and systolic and diastolic blood pressure. Body composition was assessed using a bioelectrical impedance scale (TANITA SC-330; Tanita Corporation, Tokyo, Japan). BMI was calculated as body weight divided by height squared (kg/m^2^) and classified according to the World Health Organization (WHO) criteria: normal weight, BMI < 25 kg/m^2^; excess weight (including both overweight and obesity), BMI ≥ 25 kg/m^2^, with overweight defined as BMI 25–29.9 kg/m^2^ and obesity as BMI ≥ 30 kg/m^2^ [15].

The data collection and blood analyses followed standardized criteria at Hospital Puerta de Hierro Laboratory, including centrifugation to separate plasma/serum fractions and determinations with autoanalyzer as detailed (https://investigacionpuertadehierro.com/, accessed on 11 January 2021) using validated protocols and appropriate Kits supplied by different recognized providers. Venipuncture was implemented following OMS/WHO Guidelines. Samples were stored at −80 °C until analyzed. Blood samples were obtained under fasting conditions by hospital nursing staff through venipuncture, following validated hospital protocols. Sanguineous samples were analyzed for leukocytes, lymphocytes, neutrophils, platelets, ESR and RDW determinations in the hematology laboratory of the Puerta de Hierro Majadahonda University Hospital by hospital healthcare personnel, using a SYSMEX XN-20 automated hematology analyzer (Roche, Basel, Switzerland). The routine biochemical markers such as glucose, HbA1c, total cholesterol, ferritin, triglycerides, uric acid, alanine aminotransferase (ALT), aspartate aminotransferase (AST) and transferrin were performed following standardized hospital protocols with equipment meeting accredited criteria in a quality controlled autoanalyzer (Atellica™ Solution, Siemens Healthineers, Madrid, Spain) as described elsewhere. Variables related to prognosis, proinflammatory factors and markers such as CRP, fibrinogen, insulin, lactate dehydrogenase (LDH), D-dimer, IL-6 and prothrombin activity also followed standardized procedures [16] mainly with ELISA kits (Sigma-Aldrich ELISA Kit, St. Louis, MO, USA) as described by the suppliers. TyG index (Triglyceride–Glucose index) was calculated as ln [Triglycerides (mg/dL) × Glucose (mg/dL)/2]. HOMA-IR (Homeostatic Model Assessment of Insulin Resistance) was calculated as [Fasting Glucose (mg/dL) × Fasting Insulin (µU/mL)/405]. The NLR (neutrophil-to-lymphocyte ratio) was calculated directly from the absolute neutrophil and lymphocyte counts obtained in the laboratory analyses.

### 2.3. Polygenic Risk Score Construction

DNA samples were genotyped using a high-density genome-wide array and processed externally by ADNtro (Artá, Spain). All samples passed the established quality control thresholds, each achieving a genotype call rate exceeding 99%. The PRS for SLE was calculated using the panel of lupus-associated SNPs previously identified, LD-pruned, and validated in a large GWAS/meta-analysis published by Bentham et al. [17] (Supplementary Table S2). The exact SNP set and effect sizes reported by the original authors were adopted, ensuring methodological consistency with the validated model in Europeans. All SNPs included in the PRS were directly genotyped in our dataset, eliminating the need for imputation or proxy marker selection and thereby avoiding additional LD-related uncertainty. For each individual, the PRS was computed as an additive, effect-size–weighted sum of risk alleles.

### 2.4. Statistical Analysis

Data were analyzed using R software (version 4.3.0; R Foundation for Statistical Computing, Vienna, Austria). Continuous variables are presented as mean ± standard deviation (SD) for normally distributed data, or as median [Q1–Q3] for non-normally distributed data. Normality was assessed via Shapiro–Wilk test. Between-group comparisons were performed with Student’s t test or Mann–Whitney U test, and categorical variables were analyzed using the χ^2^ test or Fisher’s exact test, as appropriate. Correlations were evaluated using Spearman’s rank coefficient. Multiple linear regression models were constructed to explore associations between PRS and metabolic/inflammatory parameters, adjusting for potential confounders (age, BMI, glucose, lipids, IL-6). Statistical significance was set at *p* < 0.05.

## 3. Results

Participants with MetS were significantly older and exhibited higher BMI, waist circumference, and visceral fat compared to those with SLE (*p* < 0.001 for all comparisons). MetS patients also had elevated systolic and diastolic blood pressure (*p* < 0.05) and fasting glucose levels (*p* < 0.001), along with a more adverse lipid profile characterized by higher triglycerides and lower HDL-C (*p* < 0.001) whereas LDL-C showed no significant differences between groups. Markers of glycemic control and insulin resistance (HbA1c, HOMA-IR) were markedly increased in MetS (*p* < 0.05), whereas albumin was slightly higher in this group (*p* = 0.002). No significant differences were observed for CRP, IL-6, or LDH. Regarding genetic predisposition, the SLE PRS was significantly higher in SLE patients, while the proinflammatory PRS did not differ significantly between groups (Table 1).

Patients with overweight/obesity showed a markedly more adverse metabolic profile compared with their normal-weight counterparts. They showed significantly higher BMI, visceral fat mass and waist circumference, together with a higher biological age estimate. In terms of biochemical parameters, overweight patients exhibited higher triglyceride levels and lower HDL cholesterol, consistent with an atherogenic lipid pattern. Fasting insulin levels were also elevated in this group, resulting in a significantly higher HOMA-IR index, indicating greater insulin resistance (Table 2).

Also, those with excess weight exhibited significantly lower TSAT levels compared to normal-weight patients (20.5% vs. 26.5%, *p* = 0.040), along with higher ESR values (15.5 mm vs. 6.0 mm, *p* = 0.025). Fibrinogen concentrations were markedly higher in the overweight/obese group (381 mg/dL vs. 295 mg/dL, *p* = 0.001). Although not reaching statistical significance, NLR and RDW showed a trend toward higher values in patients with excess weight (*p* = 0.079 and *p* = 0.076, respectively). No significant differences were observed for ferritin, leukocyte subsets, hemoglobin, platelet count or coagulation times.

Regarding genetic predisposition, the proinflammatory PRS tended to be higher in overweight/obese patients compared to normal-weight individuals, whereas the SLE-specific PRS did not differ significantly between groups.

Participants were divided into low and high SLE PRS groups. No significant differences were observed in age, BMI, body composition, blood pressure, or most biochemical parameters (*p* > 0.05). Similarly, glucose, lipid profile, liver enzymes, and inflammatory markers (CRP, IL-6) were comparable between groups. However, ferritin levels were significantly higher in the low PRS group compared to the high PRS group (*p* < 0.05). Platelet counts also differed, being higher in the low PRS group than in the high PRS group. As expected, the PRS for SLE was markedly higher in the high-risk group compared to the low-risk group (*p* < 0.001). Other hematological and coagulation markers, such as fibrinogen, D-dimer, and prothrombin time, did not show significant differences (Table 3).

**Figure 1:**
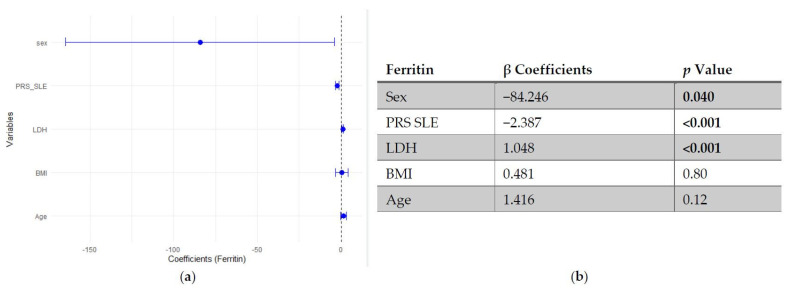
Multivariable linear regression analysis of predictors of ferritin levels: (**a**) Forest plot of β coefficients (dots) with 95% confidence intervals (horizontal lines) for sex, polygenic risk score for SLE (PRS SLE), lactate dehydrogenase (LDH), body mass index (BMI) and age. The vertical dashed line represents the null value (β = 0). (**b**) Corresponding regression estimates with 95% confidence intervals and *p*-values. β coefficients represent the expected change in ferritin concentration (ng/mL) per unit increase in the predictor, adjusted for all other variables. Sex coded as 1 = male, 2 = female; thus, negative β indicates lower ferritin in women compared to men. Four observations were excluded due to missing data. Bold numbers mean significant differences.

**Figure 2:**
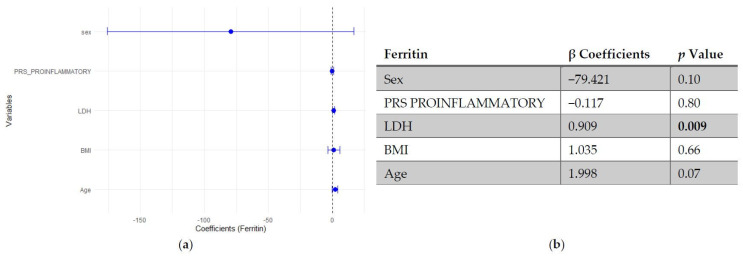
Multivariable linear regression analysis of predictors of ferritin levels: (**a**) Forest plot of β coefficients (dots) with 95% confidence intervals (horizontal lines) for sex, polygenic risk score (PRS) proinflammatory, lactate dehydrogenase (LDH), body mass index (BMI), and age. The vertical dashed line represents the null value (β = 0). (**b**) Corresponding regression estimates with 95% confidence intervals and *p*-values. β coefficients represent the expected change in ferritin concentration (ng/mL) per unit increase in the predictor, adjusted for all other variables. Sex coded as 1 = male, 2 = female; thus, negative β indicates lower ferritin in women compared to men. Four observations were excluded due to missing data. Bold numbers in the table mean significant differences.

In multivariable linear regression models adjusted for age, BMI, LDH and sex (woman), higher SLE PRS was associated with lower ferritin concentrations, while no significant association was observed for the pro-inflammatory PRS (Figure 1 and Figure 2).

**Figure 3:**
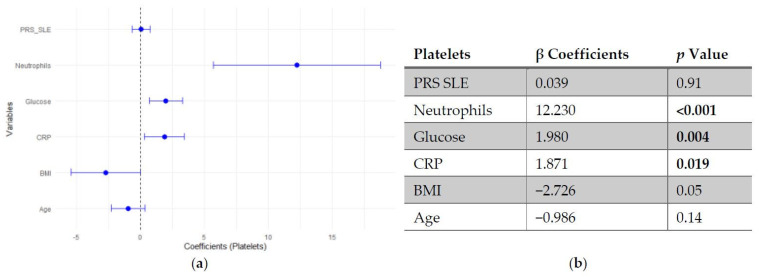
Multivariable linear regression analysis of predictors of platelets count: (**a**) Forest plot of β coefficients (dots) with 95% confidence intervals (horizontal lines) for polygenic risk score for SLE (PRS SLE), neutrophils, glucose, C-reactive protein (CRP), body mass index (BMI) and age. The vertical dashed line represents the null value (β = 0). (**b**) Corresponding regression estimates with 95% confidence intervals and *p*-values. β coefficients represent the expected change in platelets count (10^3^/μL) per unit increase in the predictor, adjusted for all other variables. Three observations were excluded due to missing data. Bold numbers in the table mean significant differences.

**Figure 4:**
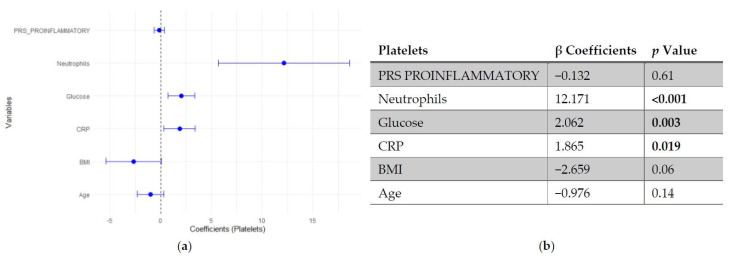
Multivariable linear regression analysis of predictors of platelets count: (**a**) Forest plot of β coefficients (dots) with 95% confidence intervals (horizontal lines) for polygenic risk score (PRS) proinflammatory, neutrophils, glucose, C-reactive protein (CRP), body mass index (BMI) and age. The vertical dashed line represents the null value (β = 0). (**b**) Corresponding regression estimates with 95% confidence intervals and *p*-values. β coefficients represent the expected change in platelets count (10^3^/μL) per unit increase in the predictor, adjusted for all other variables. Three observations were excluded due to missing data. Bold numbers in the table mean significant differences.

Regarding platelet count, neither SLE PRS nor the pro-inflammatory PRS showed significant associations with platelet count. In contrast, neutrophil count emerged as the strongest positive predictor of platelet levels, followed by fasting glucose and CRP. Age and BMI showed negative trends, though they did not reach statistical significance (Figure 3 and Figure 4).

**Figure 5:**
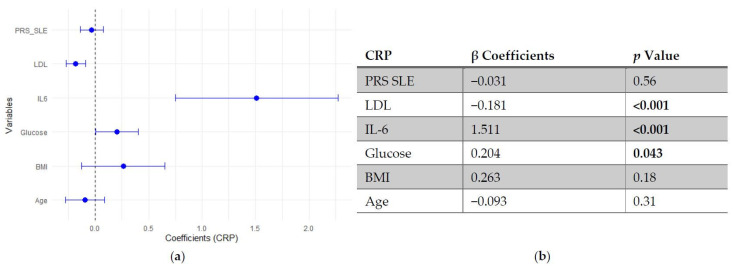
Multivariable linear regression analysis of predictors of C-reactive protein (CRP) levels: (**a**) Forest plot of β coefficients (dots) with 95% confidence intervals (horizontal lines) for polygenic risk score for SLE (PRS SLE), low-density lipoprotein (LDL), interleukin-6 (IL-6), glucose, body mass index (BMI) and age. The vertical dashed line represents the null value (β = 0). (**b**) Corresponding regression estimates with 95% confidence intervals and *p*-values. β coefficients represent the expected change in CRP levels (mg/L) per unit increase in the predictor, adjusted for all other variables. Six observations were excluded due to missing data. Bold numbers in the table mean significant differences.

**Figure 6:**
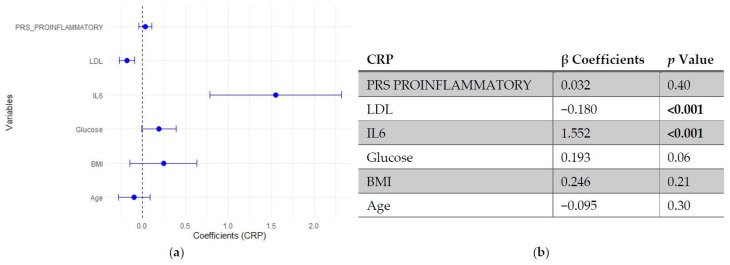
Multivariable linear regression analysis of predictors of C-reactive protein (CRP) levels: (**a**) Forest plot of β coefficients (dots) with 95% confidence intervals (horizontal lines) for polygenic risk score (PRS) proinflammatory, low-density lipoprotein (LDL), interleukin-6 (IL-6), glucose, body mass index (BMI) and age. The vertical dashed line represents the null value (β = 0). (**b**) Corresponding regression estimates with 95% confidence intervals and *p*-values. β coefficients represent the expected change in CRP levels (mg/L) per unit increase in the predictor, adjusted for all other variables. Six observations were excluded due to missing data. Bold numbers in the table mean significant differences.

In the regression models assessing predictors of CRP, neither SLE PRS nor the pro-inflammatory PRS showed significant associations with CRP levels, indicating that genetic predisposition did not independently influence this inflammatory marker. In contrast, IL-6 emerged as the strongest positive predictor of CRP, followed by fasting glucose, which showed a modest positive association. LDL cholesterol was inversely associated with CRP, while age and BMI did not reach statistical significance (Figure 5 and Figure 6).

## 4. Discussion

This study explored the relationship between genetic predisposition, assessed by PRS, and metabolic and inflammatory parameters in SLE. The analysis revealed distinct metabolic and cardiovascular risk profiles between SLE and MetS populations. It is important to note that the MetS group selected was not as a representative of the general population, but as a comparative reference characterized by chronic low-grade systemic inflammation. This design allows for disentangling disease-specific metabolic and inflammatory alterations in SLE from those driven by metabolic dysfunction alone, facilitating the identification of features uniquely associated with autoimmune processes rather than general cardiometabolic risk.

Despite a generally leaner phenotype compared with individuals with MetS, SLE patients displayed signs of possible insulin resistance (HOMA-IR > 1.97) and subtle lipid abnormalities (slightly elevated LDL levels), indicating that metabolic vulnerability can occur independently of body adiposity. In contrast, higher BMI, visceral adiposity, and waist circumference in MetS were associated with impaired glucose metabolism, reflected in slightly elevated HbA1c and HOMA-IR values. Notably, inflammatory markers such as CRP and IL-6 were comparable between groups, suggesting that systemic inflammation in SLE may occur independently of metabolic status. These findings underscore the importance of monitoring metabolic health in SLE, even in patients with low adiposity, to prevent cardiovascular complications [4,18,19,20].

In addition to conventional cardiovascular risk factors, disease-specific scoring systems may provide further insight into thromboembolic risk in SLE. For instance, the CHA_2_DS_2_-VA score has been proposed as a tool to evaluate thromboembolic risk in this population [21], offering a complementary perspective that may enhance the assessment of cardiovascular risk beyond traditional measures. Furthermore, recent frameworks in cardiometabolic evaluation emphasize the use of translational biomarkers to refine risk stratification, which may be particularly relevant in complex conditions such as SLE, where traditional indicators do not always fully capture cardiovascular vulnerability [22].

Excess adiposity in SLE was associated with a low-grade inflammatory and prothrombotic profile, as evidenced by more elevated fibrinogen and ESR levels together with reduced iron bioavailability, reflected by lower TSAT [23,24]. This pattern is consistent with a state of chronic metabolic inflammation phenotype, where iron utilization becomes impaired despite preserved or even increased ferritin levels, suggesting a shift towards an acute-phase reactant response rather than true iron deficiency [23,24,25]. The upward trends in NLR and RDW further support the presence of subclinical inflammation and oxidative stress [26], both of which have been linked to increased cardiovascular risk [27]. Altogether, these alterations highlight the potential contribution of metabolic dysregulation to cardiovascular risk stratification in overweight patients with SLE. Interestingly, when focusing on the pro-inflammatory PRS component, a trend towards higher values was observed among overweight patients, suggesting that genetic predisposition to inflammation may potentiate the metabolic-inflammatory burden associated with excess adiposity.

Within SLE population, stratification by SLE PRS indicated genetic heterogeneity, supporting PRS as a potential marker of genetic liability [4,18,19]. However, the absence of strong independent associations between PRS and most inflammatory markers underscores the multifactorial nature of SLE pathophysiology, where gene–environment interactions, treatment effects, and disease activity modulate expression of metabolic dysfunction. These findings suggest that while genetic predisposition is evident, metabolic and inflammatory variability within SLE is strongly influenced by clinical and environmental modifiers [28].

Remarkably, higher genetic risk was associated with lower ferritin levels, suggesting altered iron metabolism in genetically predisposed patients [23,24]. Moreover, individuals with high PRS exhibited lower platelet counts, indicating subtle subclinical hematological involvement consistent with the known risk of thrombocytopenia in SLE [29,30]. Recent multiomics analyses further identified *FLOT1* as a causal risk gene for SLE, with high expression in platelets and T cells, highlighting platelet-mediated mechanisms as potential contributors to disease pathogenesis [31]. These findings suggest that, even in the absence of overt clinical disease, genetic predisposition may influence iron homeostasis and platelet dynamics, emphasizing the importance of monitoring hematological parameters in genetically susceptible patients.

Recent studies further support the relevance of metabolic syndrome in rheumatic diseases. Evidence indicates that MetS and autoimmune disorders share genetic and molecular pathways, particularly those related to inflammation, oxidative stress, and metabolic signaling, suggesting the existence of overlapping pathogenic mechanisms and potentially common therapeutic targets [32]. This perspective reinforces the importance of considering metabolic dysregulation not only as a comorbidity but also as a contributing factor that may modulate disease expression in SLE.

Overall, the evidence, including this study, highlights the potential of personalized medicine in SLE through the integration of genetic, metabolic, and inflammatory profiling. Recent work has shown that individual factors such as BMI and adiposity can modulate metabolic and inflammatory signatures, including interactions with the gut microbiota [33,34]. Such interindividual variability underscores the heterogeneity of disease expression in SLE and supports a precision-based approach to patient stratification. By identifying patients with distinct immunometabolic or genetic profiles, this strategy may enhance prediction (diagnosis) and classification (prognosis) of disease, as well as guide the selection of targeted biologic therapies, optimizing treatment efficacy and minimizing adverse effects.

Among the SNVs included in the PRS for SLE, several correspond to well-established SLE susceptibility loci genes such as *IRF5*, *STAT4*, *IKZF1/2/3*, *TYK2*, *TNFAIP3*, *TNIP1*, *BLK*, *BANK1* and *PTPN22*, all consistently replicated across large GWAS meta-analyses and considered core components of the genetic architecture of SLE [4,19] (Table S2).

Our findings align with previous evidence linking metabolic syndrome components with SLE outcomes [8,9,10,11] and support the notion that metabolic dysregulation contributes to disease progression and cardiovascular risk. However, the integration of PRS data adds a new layer of interpretation: the genetic background provides a baseline risk that interacts with inflammatory and metabolic stressors rather than determining phenotype directly [4,18,19,20].

Methodologically, our work follows the principles outlined by Igo et al. (2019) [5], emphasizing that PRS should be interpreted probabilistically and contextually, considering linkage disequilibrium, population structure, and sample size limitations [7].

Strengths of the study include the comprehensive clinical characterization of participants, the use of a well-defined comparator group (MetS), and the novel integration of genetic and metabolic dimensions. Choosing MetS participants provided a relevant reference for systemic inflammation without autoimmune etiology, allowing a more nuanced assessment of the interplay between genetic predisposition, metabolic status, and inflammation in SLE. A general population cohort without metabolic disturbance might have hidden these subtle differences.

Several limitations should be considered when interpreting our findings. First, the relatively small sample size may limit statistical power and the generalizability of the results, particularly in detecting modest associations between PRS and metabolic or inflammatory markers. Second, PRS were not externally validated and the genotyping algorithm used was not fully transparent from the provider, which may affect reproducibility and robustness of the genetic risk estimates. Third, the cross-sectional design precludes causal inference, making it impossible to determine whether observed metabolic or inflammatory alterations are consequences or contributors of SLE pathophysiology. Fourth, potential confounding by treatment or disease activity was not controlled in this dataset, which could have influenced metabolic and inflammatory outcomes. Fifth, information on participant ancestry was not available, and genetic analyses were not stratified by population subgroups, which could provide insights into potential population substructure. Future studies should consider evaluating ancestry and genetic equilibrium to ensure robust interpretation of PRS across diverse populations. Finally, while metabolic and inflammatory parameters were extensively profiled, the lack of additional omics data (e.g., transcriptomics, metabolomics) constrains mechanistic insight and identification of high-risk subgroups.

Future studies should address these limitations to enhance the understanding of immunometabolic pathways in SLE and help identify subgroups at higher cardiometabolic risk.

## 5. Conclusions

Polygenic risk scores derived from lupus-associated SNVs revealed genetic interindividual variability within SLE patients and discriminated them from a metabolic syndrome reference group. Although PRS SLE showed a significant association with ferritin levels, its contribution to broader metabolic and inflammatory variability was limited. Importantly, SLE patients exhibited metabolic and inflammatory alterations that may increase cardiovascular risk, underscoring the need for careful cardiometabolic monitoring. These results suggest that genetic predisposition contributes to, but does not fully explain, the metabolic heterogeneity of SLE. Integrating PRS into clinical and biochemical profiling may improve personalized risk assessment and understanding of autoimmune pathophysiology for precision management.

## Figures and Tables

**Table 1:** Clinical, anthropometric, body composition and biochemical variables in systemic lupus erythematosus (SLE) and metabolic syndrome (MetS) populations.

	Overall	SLE	MetS	P Test
*n*	93	56	37	
Clinical, anthropometric and body composition measurements				
Age (years) ^a^	56.9 (11.7)	53.2 (12.3)	62.5 (8.3)	**<0.001**
Sex (women) ^b^	67 (72.0)	51 (91.1)	16 (43.2)	**<0.001**
BMI (kg/m^2^) ^a^	29.70 (5.70)	27.79 (5.74)	32.58 (4.28)	**<0.001**
Total muscle mass (kg) ^c^	44.8 [39.9, 53.2]	42.7 [39.0, 46.3]	52.1 [44.8, 62.5]	**<0.001**
Total fat mass (%) ^c^	36.1 [28.8, 42.6]	34.7 [27.6, 41.6]	37.6 [31.5, 44.2]	0.21
Visceral fat (AU) ^a^	11.37 (5.46)	8.71 (4.34)	15.39 (4.44)	**<0.001**
Biological age (years) ^c^	63.0 [48.0, 74.0]	53.5 [41.75, 66.0]	70.0 [63.0, 78.0]	**<0.001**
Waist circumference (cm) ^a^	102.2 (14.3)	96.5 (13.9)	110.8 (9.7)	**<0.001**
WHR ^a^	0.95 (0.09)	0.91 (0.07)	1.00 (0.08)	**<0.001**
SBP (mmHg) ^c^	131.0 [118.0, 147.0]	125.0 [115.0, 140.25]	141.0 [127.0, 150.0]	**0.001**
DBP (mmHg) ^a^	77.7 (11.5)	75.3 (11.9)	81.3 (9.9)	**0.012**
Biochemical data				
Glucose (mg/dL) ^c^	94.0 [88.0, 103.75]	90.0 [84.0, 95.0]	101.0 [95.0, 108.5]	**<0.001**
Albumin (g/dL) ^c^	4.50 [4.32, 4.68]	4.50 [4.30, 4.60]	4.60 [4.50, 4.80]	**0.002**
Uric (mg/dL) ^a^	5.28 (1.47)	4.84 (1.40)	5.94 (1.35)	**<0.001**
Total cholesterol (mg/dL) ^a^	175.69 (35.79)	181.87 (34.93)	166.42 (35.52)	**0.044**
LDL (mg/dL) ^a^	99.37 (30.21)	102.45 (27.65)	94.83 (33.50)	0.25
HDL (mg/dL) ^c^	53.00 [42.00, 64.00]	59.00 [51.00, 71.00]	44.50 [38.75, 50.50]	**<0.001**
Triglycerides (mg/dL) ^c^	106.0 [80.0, 134.25]	92.5 [67.0, 126.0]	109.5 [93.75, 170.5]	**0.011**
TyG (mg/dL ) ^a^	8.50 (0.52)	8.35 (0.53)	8.74 (0.40)	**<0.001**
HbA1c (%) ^c^	5.50 [5.20, 5.80]	5.40 [5.10, 5.70]	5.75 [5.40, 6.03]	**0.001**
Insulin (μIU/mL) ^c^	8.78 [6.27, 12.13]	8.75 [4.95, 11.48]	10.26 [6.91, 16.10]	0.11
HOMA-IR ^c^	2.19 [1.38, 2.95]	2.07 [1.09, 2.61]	2.41 [1.74, 4.07]	**0.014**
Bilirubin (mg/dL) ^c^	0.60 [0.50, 0.80]	0.60 [0.50, 0.70]	0.75 [0.58, 1.12]	**0.007**
GOT/AST (U/L) ^c^	22.5 [19.0, 26.7]	21.0 [18.0, 26.0]	24.5 [20.0, 28.5]	0.08
GPT/ALT (U/L) ^c^	21.0 [17.0, 32.0]	18.0 [15.0, 25.0]	28.0 [22.5, 38.0]	**<0.001**
GGT (U/L) ^c^	19.5 [15.0, 33.0]	17.0 [13.0, 25.0]	25.0 [17.75, 36.25]	**0.003**
ALP (U/L) ^c^	72.0 [55.25, 84.75]	68.5 [55.0, 84.75]	72.0 [62.75, 80.5]	0.57
LDH (U/L) ^c^	188.0 [162.25, 204.75]	192.5 [171.25, 208.0]	180.0 [159.25, 204.0]	0.29
CRP (mg/L) ^c^	1.4 [0.9, 5.5]	1.0 [0.9, 5.0]	2.0 [0.9, 5.75]	0.39
IL-6 (pg/mL) ^c^	2.5 [2.5, 3.58]	2.5 [2.5, 3.45]	2.5 [2.5, 3.65]	0.65
Ferritin (ng/mL) ^c^	81.0 [48.0, 170.0]	57.5 [40.25, 132.0]	154.0 [83.5, 245.0]	**<0.001**
Leukocytes (10^3^/μL) ^c^	5.91 [4.74, 7.19]	5.18 [4.32, 6.59]	6.62 [5.46, 7.75]	**0.007**
Neutrophils (10^3^/μL) ^c^	3.60 [2.63, 4.70]	3.39 [2.27, 4.71]	3.75 [3.30, 4.50]	0.08
Lymphocytes (10^3^/μL) ^c^	1.58 [1.30, 1.90]	1.40 [1.10, 1.70]	1.80 [1.57, 2.42]	**<0.001**
NLR ^c^	2.03 [1.60, 3.27]	2.42 [1.52, 3.58]	1.87 [1.64, 2.44]	0.10
Monocytes (10^3^/μL) ^c^	0.40 [0.30, 0.50]	0.30 [0.29, 0.40]	0.41 [0.37, 0.50]	**0.011**
Platelets (10^3^/μL) ^c^	229.0 [193.0, 273.0]	218.0 [185.0, 273.0]	233.0 [202.25, 270.0]	0.50
Hemoglobin (g/dL) ^a^	14.64 (1.29)	14.23 (1.05)	15.25 (1.40)	**<0.001**
Hematocrit (%) ^c^	43.20 [41.28, 45.92]	42.45 [41.02, 44.30]	45.90 [43.20, 48.52]	**<0.001**
Fibrinogen (mg/dL) ^c^	345.0 [296.0, 404.0]	348.5 [291.0, 396.5]	345.0 [309.0, 418.0]	0.39
D-dimer (ng/mL) ^c^	312.0 [224.25, 469.75]	312.0 [214.75, 504.0]	318.50 [244.25, 438.5]	0.96
Polygenic risk score (PRS)				
PRS proinflammatory ^c^	45.0 [31.0, 82.0]	51.0 [37.0, 85.25]	43.0 [12.0, 68.0]	0.10
PRS SLE ^c^	88.0 [63.0, 97.0]	93.5 [74.75, 98.25]	73.0 [51.0, 92.0]	**0.005**

Raw data. ^a^ Data shown as mean and standard deviation (SD). ^b^ Data shown as number of cases and frequency (%). ^c^ Data shown as median [Q1–Q3]. Between-group comparisons were performed using Student’s *t*-test for normally distributed variables and the Mann–Whitney U test for non-normally distributed variables. Categorical variables were compared using the chi-squared test. Bold numbers mean significant differences. BMI: Body Mass Index. BMR: basal metabolic rate. WHR: waist-hip ratio. SBP: systolic blood pressure. DBP: diastolic blood pressure. LDL: low-density lipoprotein. HDL: high-density lipoprotein. TyG index (Triglyceride–Glucose index) was calculated as ln [Triglycerides (mg/dL) × Glucose (mg/dL)/2]. HbA1c: glycosylated hemoglobin. HOMA-IR (Homeostatic Model Assessment of Insulin Resistance) was calculated as [Fasting Glucose (mg/dL) × Fasting Insulin (µU/mL)/405]. AST: aspartate aminotransferase. ALT: alanine aminotransferase. GGT: gamma-glutamyl transferase. ALP: alkaline phosphatase. LDH: lactate dehydrogenase. CRP: C-reactive protein. IL-6: interleukin-6. NLR: neutrophil-to-lymphocyte ratio.

**Table 2:** Clinical, anthropometric, body composition and biochemical variables in systemic lupus erythematosus (SLE) populations (normal weight and excess weight).

	Overall	SLE Normal Weight	SLE Excess Weight	P Test
*n*	56	21	35	
Clinical, anthropometric and body composition measurements				
Age (years) ^a^	53.2 (12.3)	49.5 (11.9)	55.4 (12.1)	0.08
Sex (women) ^b^	51 (91.1)	21 (100.0)	30 (85.7)	0.18
BMI (kg/m^2^) ^a^	27.79 (5.74)	22.36 (1.92)	31.05 (4.69)	**<0.001**
Total muscle mass (kg) ^c^	42.7 [38.9, 46.3]	39.9 [38.3, 44.0]	44.6 [40.9, 48.4]	**0.010**
Total fat mass (%) ^c^	34.7 [27.6, 41.6]	27.4 [24.9, 30.5]	40.1 [35.6, 46.4]	**<0.001**
Visceral fat (AU) ^a^	8.71 (4.34)	4.71 (1.85)	11.10 (3.57)	**<0.001**
Biological age (years) ^c^	53.5 [41.75, 66.00]	38.0 [30.00, 43.00]	64.0 [56.50, 75.00]	**<0.001**
Waist circumference (cm) ^a^	96.7 (13.9)	83.0 (5.9)	104.5 (10.7)	**<0.001**
WHR ^a^	0.91 (0.07)	0.86 (0.05)	0.95 (0.07)	**<0.001**
SBP (mmHg) ^c^	125.0 [115.0, 140.25]	118.0 [113.0, 126.0]	131.0 [120.0, 147.5]	**0.001**
DBP (mmHg) ^a^	75.3 (11.9)	67.5 (9.5)	79.9 (10.7)	**<0.001**
Biochemical data				
Glucose (mg/dL) ^c^	90.0 [84.0, 95.0]	86.0 [81.75, 90.0]	93.0 [88.0, 97.75]	**0.009**
Albumin (g/dL) ^c^	4.50 [4.30, 4.60]	4.50 [4.30, 4.60]	4.50 [4.30, 4.60]	0.81
Uric (mg/dL) ^a^	4.84 (1.40)	4.24 (1.53)	5.20 (1.20)	**0.012**
Total cholesterol (mg/dL) ^a^	181.87 (34.93)	197.25 (25.71)	172.82 (36.76)	**0.012**
LDL (mg/dL) ^a^	102.45 (27.65)	112.75 (22.07)	96.21 (29.10)	**0.033**
HDL (mg/dL) ^c^	59.0 [51.0, 71.0]	63.0 [57.5, 79.75]	56.0 [43.0, 65.0]	**0.016**
Triglycerides (mg/dL) ^c^	92.5 [67.0, 126.0]	85.5 [58.0, 99.0]	117.5 [69.75, 136.5]	**0.025**
TyG (mg/dL) ^a^	8.35 (0.53)	8.11 (0.46)	8.49 (0.52)	**0.010**
HbA1c (%) ^c^	5.40 [5.10, 5.70]	5.15 [5.00, 5.50]	5.50 [5.20, 5.70]	**0.006**
Insulin (μIU/mL) ^c^	8.75 [4.95, 11.48]	6.64 [3.67, 9.41]	9.80 [7.08, 13.32]	**0.008**
HOMA-IR ^c^	2.07 [1.09, 2.61]	1.32 [0.77, 2.09]	2.26 [1.48, 2.93]	**0.003**
GOT/AST (U/L) ^c^	21.0 [18.0, 26.0]	21.0 [17.75, 23.75]	22.0 [18.25, 26.0]	0.54
GPT/ALT (U/L) ^c^	18.0 [15.0, 25.0]	15.5 [13.75, 18.25]	20.5 [17.0, 26.5]	**0.010**
GGT (U/L) ^c^	17.0 [13.0, 25.0]	14.5 [12.0, 19.25]	19.5 [14.25, 28.75]	**0.027**
ALP (U/L) ^c^	68.5 [55.0, 84.75]	62.5 [55.0, 69.0]	78.0 [55.0, 89.5]	**0.036**
LDH (U/L) ^c^	192.5 [171.25, 208.0]	176.5 [156.5, 198.5]	197.0 [179.5, 219.0]	**0.021**
CRP (mg/L) ^c^	1.00 [0.90, 5.00]	0.90 [0.73, 0.92]	2.25 [0.90, 9.40]	**0.002**
IL-6 (pg/mL) ^c^	2.50 [2.50, 3.45]	2.50 [2.50, 2.95]	2.80 [2.50, 4.60]	0.15
Ferritin (ng/mL) ^c^	57.50 [40.25, 132.00]	53.50 [30.25, 95.00]	70.50 [45.75, 132.00]	0.38
TSAT (%) ^c^	23.0 [18.0, 29.25]	26.5 [22.5, 34.75]	20.5 [16.75, 26.25]	**0.040**
Leukocytes (10^3^/μL) ^c^	5.18 [4.32, 6.59]	4.89 [4.22, 6.95]	5.21 [4.52, 6.26]	0.75
Neutrophils (10^3^/μL) ^c^	3.39 [2.27, 4.71]	2.80 [1.97, 4.18]	3.41 [2.67, 4.71]	0.31
Lymphocytes (10^3^/μL) ^c^	1.40 [1.10, 1.70]	1.54 [1.10, 1.75]	1.30 [1.12, 1.67]	0.26
NLR ^c^	2.42 [1.52, 3.58]	1.81 [1.33, 3.08]	2.63 [1.74, 3.77]	0.08
Monocytes (10^3^/μL) ^c^	0.30 [0.29, 0.40]	0.30 [0.30, 0.40]	0.31 [0.27, 0.42]	1.00
Platelets (10^3^/μL) ^c^	218.0 [185.0, 273.0]	237.0 [191.75, 275.5]	207.0 [185.0, 264.0]	0.68
Hemoglobin (g/dL) ^a^	14.23 (1.05)	14.14 (1.00)	14.29 (1.08)	0.64
Hematocrit (%) ^c^	42.45 [41.02, 44.30]	42.60 [40.95, 43.28]	42.35 [41.10, 44.55]	0.67
MCV (fL) ^a^	91.95 (3.83)	91.19 (2.43)	92.40 (4.42)	0.27
MCHC (g/dL) ^c^	33.50 [32.80, 33.90]	33.55 [33.20, 33.82]	33.45 [32.52, 34.05]	0.64
RDW (fL) ^c^	13.60 [13.03, 14.78]	13.40 [12.90, 13.93]	13.95 [13.12, 15.10]	0.08
ESR (mm) ^c^	11.0 [4.0, 20.0]	6.0 [3.0, 11.5]	15.5 [6.0, 21.75]	**0.025**
Prothrombin Time ^c^	98.0 [89.0, 108.75]	97.0 [92.75, 102.50]	103.0 [87.75, 108.75]	0.91
aPTT (s) ^c^	30.90 [28.98, 35.12]	31.90 [29.73, 35.23]	30.60 [28.90, 33.90]	0.40
Fibrinogen (mg/dL) ^c^	348.5 [291.0, 396.5]	295.5 [246.0, 354.75]	381.0 [314.25, 440.0]	**0.001**
D-dimer (ng/mL) ^c^	312.0 [214.75, 504.0]	290.0 [221.5, 358.25]	360.5 [209.75, 618.25]	0.49
Polygenic risk score (PRS)				
PRS proinflammatory ^c^	51.0 [37.0, 85.25]	43.0 [37.0, 57.0]	74.0 [34.0, 87.5]	0.07
PRS SLE ^c^	93.5 [74.75, 98.25]	88.0 [73.0, 99.0]	94.0 [76.5, 98.0]	0.86

Raw data, adjusted for age and sex. ^a^ Data shown as mean and standard deviation (SD). ^b^ Data shown as number of cases and frequency (%). ^c^ Data shown as median [Q1–Q3]. Between-group comparisons were performed using age-adjusted linear models for continuous variables and age-adjusted logistic models for categorical variables. Bold numbers mean significant differences. BMI: Body Mass Index. WHR: waist-hip ratio. SBP: systolic blood pressure. DBP: diastolic blood pressure. LDL: low-density lipoprotein. HDL: high-density lipoprotein. TyG index (Triglyceride–Glucose index) was calculated as ln [Triglycerides (mg/dL) × Glucose (mg/dL)/2]. HbA1c: glycosylated hemoglobin. HOMA-IR (Homeostatic Model Assessment of Insulin Resistance) was calculated as [Fasting Glucose (mg/dL) × Fasting Insulin (µU/mL)/405]. AST: aspartate aminotransferase. ALT: alanine aminotransferase. GGT: gamma-glutamyl transferase. ALP: alkaline phosphatase. LDH: lactate dehydrogenase. CRP: C-reactive protein. IL-6: interleukin-6. TSAT: transferrin saturation index. NLR: neutrophil-to-lymphocyte ratio. MCV: mean corpuscular volume. MCHC: mean corpuscular hemoglobin concentration. RDW: red cell distribution width. ESR: erythrocyte sedimentation rate. aPTT: activated partial thromboplastin time.

**Table 3:** SLE population stratified by median PRS.

	Overall	Low SLE PRS	High SLE PRS	P Test
*n*	56	28	28	
Clinical, anthropometric and body composition measurements				
Age (years) ^a^	53.18 (12.26)	55.00 (10.92)	51.36 (13.42)	0.27
Sex (women) ^b^	51 (91.1)	25 (89.3)	26 (92.9)	1.00
BMI (kg/m^2^) ^a^	27.79 (5.74)	27.68 (5.87)	27.90 (5.71)	0.89
Total muscle mass (kg) ^c^	42.65 [38.98, 46.32]	40.90 [37.88, 45.75]	42.90 [39.75, 46.32]	0.44
Total fat mass (%) ^c^	34.65 [27.55, 41.62]	34.10 [27.37, 44.58]	35.60 [29.42, 41.23]	0.86
Visceral fat (AU) ^a^	8.71 (4.34)	9.00 (4.46)	8.41 (4.29)	0.62
Biological age (years) ^c^	53.5 [41.75, 66.00]	57.5 [41.00, 67.50]	49.0 [41.75, 64.25]	0.81
Body water (%) ^c^	46.45 [42.32, 51.47]	46.85 [42.27, 52.55]	46.45 [42.55, 51.05]	0.69
Waist circumference (cm) ^a^	96.46 (13.96)	95.43 (14.26)	97.50 (13.83)	0.58
WHR ^a^	0.91 (0.07)	0.91 (0.07)	0.92 (0.08)	0.50
SBP (mmHg) ^c^	125.00 [115.00, 140.25]	129.50 [114.75, 141.50]	120.50 [116.50, 129.75]	0.26
DBP (mmHg) ^a^	75.27 (11.86)	75.25 (14.25)	75.29 (9.13)	0.99
Biochemical data				
Glucose (mg/dL) ^c^	90.00 [84.00, 95.00]	91.00 [85.00, 96.00]	88.50 [82.25, 93.75]	0.20
Albumin (g/dL) ^c^	4.50 [4.30, 4.60]	4.50 [4.30, 4.60]	4.40 [4.30, 4.57]	0.15
Total cholesterol (mg/dL) ^a^	181.87 (34.93)	187.04 (30.08)	176.31 (39.34)	0.26
LDL (mg/dL) ^a^	102.45 (27.65)	107.07 (25.93)	97.65 (29.05)	0.22
HDL (mg/dL) ^c^	59.00 [51.00, 71.00]	61.00 [54.00, 71.00]	55.50 [45.75, 69.00]	0.58
Triglycerides (mg/dL) ^c^	92.50 [67.00, 126.00]	88.00 [71.25, 125.25]	101.00 [66.25, 127.50]	0.97
HbA1c (%) ^c^	5.40 [5.10, 5.70]	5.50 [5.15, 5.70]	5.35 [5.07, 5.62]	0.43
Insulin (μIU/mL) ^c^	8.75 [4.95, 11.48]	8.81 [4.04, 10.88]	8.62 [5.72, 12.92]	0.33
Bilirubin (mg/dL) ^c^	0.60 [0.50, 0.70]	0.60 [0.50, 0.80]	0.50 [0.40, 0.60]	**0.033**
GOT/AST (U/L) ^c^	21.0 [18.0, 26.0]	23.0 [19.75, 26.0]	20.5 [17.0, 24.0]	0.26
GPT/ALT (U/L) ^c^	18.0 [15.0, 25.0]	17.5 [15.75, 26.0]	18.0 [15.0, 25.0]	0.71
GGT (U/L) ^c^	17.0 [13.0, 25.0]	19.0 [13.0, 29.0]	16.0 [13.25, 24.5]	0.84
ALP (U/L) ^c^	68.5 [55.0, 84.75]	64.0 [54.5, 83.25]	73.0 [57.0, 86.0]	0.51
Urea (mg/dL) ^c^	34.5 [29.0, 40.75]	35.0 [30.75, 39.25]	31.5 [28.0, 43.75]	0.49
Creatinine (mg/dL) ^c^	0.78 [0.70, 0.86]	0.78 [0.69, 0.84]	0.80 [0.72, 0.86]	0.86
LDH (U/L) ^c^	192.5 [171.25, 208.0]	188.0 [169.0, 198.75]	198.0 [179.0, 219.75]	0.12
CRP (mg/L) ^c^	1.00 [0.90, 5.00]	0.95 [0.90, 4.25]	1.20 [0.90, 6.50]	0.46
IL-6 (pg/mL) ^c^	2.50 [2.50, 3.45]	2.50 [2.50, 3.20]	2.50 [2.50, 4.57]	0.60
Ferritin (ng/mL) ^c^	57.50 [40.25, 132.00]	77.00 [49.50, 155.00]	49.00 [28.00, 81.00]	**0.020**
Folic Acid (ng/mL) ^c^	7.10 [5.70, 13.20]	7.50 [5.77, 13.32]	7.10 [5.50, 13.10]	0.99
Leukocytes (10^3^/μL) ^c^	5.18 [4.32, 6.59]	5.02 [4.40, 6.95]	5.18 [3.77, 6.30]	0.56
Neutrophils (10^3^/μL) ^c^	3.39 [2.27, 4.71]	3.45 [2.49, 5.03]	3.34 [2.00, 4.13]	0.30
Lymphocytes (10^3^/μL) ^c^	1.40 [1.10, 1.70]	1.50 [1.16, 1.70]	1.31 [1.10, 1.60]	0.44
Monocytes (10^3^/μL) ^c^	0.30 [0.29, 0.40]	0.30 [0.28, 0.41]	0.30 [0.30, 0.40]	0.95
Platelets (10^3^/μL) ^c^	218.0 [185.0, 273.0]	245.0 [200.25, 283.75]	203.0 [180.0, 240.0]	0.05
Hemoglobin (g/dL) ^a^	14.23 (1.05)	14.35 (1.13)	14.10 (0.95)	0.39
Hematocrit (%) ^c^	42.45 [41.02, 44.30]	42.25 [40.88, 43.98]	42.75 [41.30, 44.30]	0.80
MCV (fL) ^a^	91.95 (3.83)	91.68 (3.37)	92.25 (4.31)	0.59
MCHC (g/dL) ^c^	33.50 [32.80, 33.90]	33.60 [33.27, 33.95]	33.25 [32.52, 33.88]	0.35
RDW (fL) ^c^	13.60 [13.03, 14.78]	13.55 [13.00, 14.48]	13.95 [13.25, 15.28]	0.37
ESR (mm) ^c^	11.0 [4.0, 20.0]	11.0 [3.0, 29.5]	12.0 [6.0, 19.0]	0.74
Prothrombin Time ^c^	98.0 [89.0, 108.75]	97.0 [88.0, 108.75]	99.0 [90.75, 108.0]	0.75
aPTT (s) ^c^	30.90 [28.98, 35.12]	31.20 [28.90, 34.52]	30.65 [29.23, 35.15]	0.91
Fibrinogen (mg/dL) ^c^	348.5 [291.0, 396.5]	344.0 [288.0, 408.0]	348.5 [296.0, 380.5]	0.99
D-dimer (ng/mL) ^c^	312.0 [214.75, 504.0]	277.0 [203.5, 433.5]	350.0 [227.5, 549.5]	0.31
Polygenic risk score (PRS)				
PRS proinflammatory ^c^	51.0 [37.0, 85.25]	54.0 [37.0, 86.0]	47.0 [31.0, 82.0]	0.54
PRS SLE ^c^	93.5 [74.75, 98.25]	74.5 [58.75, 86.0]	98.5 [97.0, 99.0]	**<0.001**

Raw data, adjusted for age and BMI. ^a^ Data shown as mean and standard deviation (SD). ^b^ Data shown as number of cases and frequency (%). ^c^ Data shown as median [Q1–Q3]. Participants were divided into “Low” and “High” groups based on the median PRS SLE value (93.5). Between-group comparisons were performed using linear models adjusted for age and BMI for continuous variables, and logistic models adjusted for age and BMI for categorical variables. Bold numbers mean significant differences. BMI: Body Mass Index. WHR: waist-hip ratio. SBP: systolic blood pressure. DBP: diastolic blood pressure. LDL: low-density lipoprotein. HDL: high-density lipoprotein. HbA1c: glycosylated hemoglobin. AST: aspartate aminotransferase. ALT: alanine aminotransferase. GGT: gamma-glutamyl transferase. ALP: alkaline phosphatase. LDH: lactate dehydrogenase. CRP: C-reactive protein. IL-6: interleukin-6. MCV: mean corpuscular volume. MCHC: mean corpuscular hemoglobin concentration. RDW: red cell distribution width. ESR: erythrocyte sedimentation rate. aPTT: activated partial thromboplastin time.

## Data Availability

Data are available upon reasonable request to the authors.

## Supplementary Materials

The following supporting information can be downloaded at: https://www.mdpi.com/article/10.3390/genes17010053/s1, Table S1: Polygenic risk score (PRS) in systemic lupus erythematosus (SLE) and metabolic syndrome (MS) populations; Table S2: Systemic lupus erythematosus (SLE) single nucleotide polymorphisms (SNPs) for polygenic risk score (PRS).

## Abbreviations

The following abbreviations are used in this manuscript:

ALP	Alkaline phosphatase
ALT	Alanine aminotransferase
aPTT	Activated partial thromboplastin time
AST	Aspartate aminotransferase
BMI	Body mass index
BMR	Basal metabolic rate
CRP	C-reactive protein
DBP	Diastolic blood pressure
ESR	Erythrocyte sedimentation rate
GGT	Gamma-glutamyl transferase
GWAS	Genome-wide association studies
HbA1c	Glycosylated hemoglobin
HDL	High-density lipoprotein
HOMA-IR	Homeostatic Model Assessment of Insulin Resistance
IL-6	Interleukin-6
IQR	Interquartile range
LDH	Lactate dehydrogenase
LDL	Low-density lipoprotein
MCHC	Mean corpuscular hemoglobin concentration
MCV	Mean corpuscular volume
MetS	Metabolic syndrome
NLR	Neutrophil-to-lymphocyte ratio
PRS	Polygenic risk score
RDW	Red cell distribution width
SBP	Systolic blood pressure
SD	Standard deviation
SLE	Systemic lupus erythematosus
SNVs	Single nucleotide variants
TyG	Triglyceride-glucose
WHR	Waist-hip ratio

## References

[1] Tsokos G.C. (2011). Systemic lupus erythematosus. N. Engl. J. Med..

[2] Kaul A., Gordon C., Crow M.K., Touma Z., Urowitz M.B., van Vollenhoven R., Ruiz-Irastorza G., Hughes G. (2016). Systemic lupus erythematosus. Nat. Rev. Dis. Primers.

[3] Fava A., Petri M. (2019). Systemic lupus erythematosus: Diagnosis and clinical management. J. Autoimmun..

[4] Laurynenka V., Harley J.B. (2024). The 330 risk loci known for systemic lupus erythematosus (SLE): A review. Front. Lupus.

[5] Igo R.P., Kinzy T.G., Cooke Bailey J.N. (2019). Genetic Risk Scores. Curr. Protoc. Hum. Genet..

[6] Xu B., Liu Y., Chen G., Jiang P., Qu Y., Wang M., Kao X. (2025). Genome-wide analysis of abnormal splicing regulators and alternative splicing involved in immune regulation in systemic lupus erythematosus. Autoimmunity.

[7] Chung C.P., Avalos I., Oeser A., Gebretsadik T., Shintani A., Raggi P., Stein C.M. (2007). High prevalence of the metabolic syndrome in patients with systemic lupus erythematosus: Association with disease characteristics and cardiovascular risk factors. Ann. Rheum. Dis..

[8] Parker B., Urowitz M.B., Gladman D.D., Lunt M., Donn R., Bae S.C., Sanchez-Guerrero J., Romero-Diaz J., Gordon C., Wallace D.J. (2015). Impact of early disease factors on metabolic syndrome in systemic lupus erythematosus: Data from an international inception cohort. Ann. Rheum. Dis..

[9] Terrell M., Morel L. (2022). The Intersection of Cellular and Systemic Metabolism: Metabolic Syndrome in Systemic Lupus Erythematosus. Endocrinology.

[10] Hotamisligil G.S. (2017). Inflammation, metaflammation and immunometabolic disorders. Nature.

[11] Esser N., Legrand-Poels S., Piette J., Scheen A.J., Paquot N. (2014). Inflammation as a link between obesity, metabolic syndrome and type 2 diabetes. Diabetes Res. Clin. Pract..

[12] Xu Z., Yang S., Tan Y., Zhang Q., Wang H., Tao J., Liu Q., Wang Q., Feng W., Li Z. (2025). Inflammation in cardiovascular-kidney-metabolic syndrome: Key roles and underlying mechanisms-a comprehensive review. Mol. Cell. Biochem..

[13] Aringer M., Costenbader K., Daikh D., Brinks R., Mosca M., Ramsey-Goldman R., Smolen J.S., Wofsy D., Boumpas D.T., Kamen D.L. (2019). 2019 European League Against Rheumatism/American College of Rheumatology Classification Criteria for Systemic Lupus Erythematosus. Arthritis Rheumatol..

[14] Lorenzo C., Williams K., Hunt K.J., Haffner S.M. (2007). The National Cholesterol Education Program—Adult Treatment Panel III, International Diabetes Federation, and World Health Organization definitions of the metabolic syndrome as predictors of incident cardiovascular disease and diabetes. Diabetes Care.

[15] (OMS) OMdlS Obesidad y Sobrepeso Disponible en. https://www.who.int/es/news-room/fact-sheets/detail/obesity-and-overweight.

[16] Moreno-Torres V., Castejón R., Martínez-Urbistondo M., Gutiérrez-Rojas Á., Vázquez-Comendador J., Tutor P., Campo P.D., Mellor-Pita S., Rosado S., Vargas-Núñez J. (2022). Serum cytokines to predict systemic lupus erythematosus clinical and serological activity. Clin. Transl. Sci..

[17] Bentham J., Morris D.L., Graham D.S.C., Pinder C.L., Tombleson P., Behrens T.W., Martín J., Fairfax B.P., Knight J.C., Chen L. (2015). Genetic association analyses implicate aberrant regulation of innate and adaptive immunity genes in the pathogenesis of systemic lupus erythematosus. Nat. Genet..

[18] Chen Y.C., Liu T.Y., Lu H.F., Huang C.M., Liao C.C., Tsai F.J. (2024). Multiple polygenic risk scores can improve the prediction of systemic lupus erythematosus in Taiwan. Lupus Sci. Med..

[19] Khunsriraksakul C., Markus H., Olsen N.J., Carrel L., Jiang B., Liu D.J. (2022). Construction and Application of Polygenic Risk Scores in Autoimmune Diseases. Front. Immunol..

[20] Mok C.C. (2019). Metabolic syndrome and systemic lupus erythematosus: The connection. Expert Rev. Clin. Immunol..

[21] Dziedzic R., Węgiel M., Siwiec-Koźlik A., Spałkowska M., Zaręba L., Bazan-Socha S., Korkosz M., Kosałka-Węgiel J. (2025). Thromboembolic Episodes in Patients with Systemic Lupus Erythematosus Without Atrial Fibrillation/Atrial Flutter Are Related to the Presence of at Least 3 Points in the CHA. J. Clin. Med..

[22] Visioli F., Urbistondo D.M., Gkipalis S., Vidal-Ostos De Lara F., Ruiz-Saavedra A., Leon M., Chaib F.B., Hernández A.H., Acha M.F.L., Laparra M. (2025). Translational biomarkers for integrated cardiovascular disease risk assessment: A multidisciplinary review with applications in precision medicine. Nutr. Metab. Cardiovasc. Dis..

[23] Li N., Chai R., Xu X., Xu Q., Liang J., Wang D., Zhang H., Feng X., Geng L., Sun L. (2025). Serum untargeted metabolomics alterations in systemic lupus erythematosus patients with elevated serum ferritin. Sci. Rep..

[24] Mittal S., Agarwal P., Wakhlu A., Kumar A., Mehrotra R. (2016). Anaemia in Systemic Lupus Erythematosus Based on Iron Studies and Soluble Transferrin Receptor Levels. J. Clin. Diagn. Res..

[25] Zhao G., Li X., Zhang Y., Wang X., Deng L., Xu J., Jin S., Zuo Z., Xun L., Luo M. (2025). Intricating connections: The role of ferroptosis in systemic lupus erythematosus. Front. Immunol..

[26] Lao X., Ma L., Ma Q., Yang Z., Guo L., Nong W. (2020). Hematological factors associated with immunity, inflammation, and metabolism in patients with systemic lupus erythematosus: Data from a Zhuang cohort in Southwest China. J. Clin. Lab. Anal..

[27] González-Sierra M., Romo-Cordero A., Quevedo-Abeledo J.C., Quevedo-Rodríguez A., Gómez-Bernal F., de Vera-González A., López-Mejías R., Martín-González C., González-Gay M.Á., Ferraz-Amaro I. (2023). Red Cell Distribution Width Association with Subclinical Cardiovascular Disease in Patients with Rheumatoid Arthritis. J. Clin. Med..

[28] Martínez-Urbistondo M., Higuera-Gómez A., de Cuevillas B., Cuevas-Sierra A., Mellor-Pita S., Moreno-Torres V., Vargas J.-A., Castejón R., Martínez J.A. (2025). Visceral fat, cardiovascular risk factors and quality of life in lupus activity categorised via complement C3. Lupus Sci. Med..

[29] Pamuk O.N. (2023). Thrombocytopenia in Patients with Systemic Lupus Erythematosus. Eur. J. Rheumatol..

[30] Zheng Z., Liu J., Yun M., Deng L., Xiang P., Jiang M., Wang R., Liu C. (2025). Immune thrombocytopenia in patients with systemic lupus erythematosus. Clin. Rheumatol..

[31] Fu L., Yu J., Wang X., Chen Z., Sun J., Gao F., Zhang Z., Fu J., Hong P., Feng W. (2025). Investigating Overlapping Genetic Factors and Novel Causal Genes in Autoimmune Diseases: A Transcriptome-Wide Association and Multiomics Study. Int. J. Genom..

[32] Shi Y., Guan S., Liu X., Zhai H., Zhang Y., Liu J., Yang W., Wang Z. (2025). Genetic Commonalities Between Metabolic Syndrome and Rheumatic Diseases Through Disease Interactome Modules. J. Cell Mol. Med..

[33] Chero-Sandoval L., Higuera-Gómez A., Cuevas-Sierra A., de Cuevillas B., Castejón R., Martínez-Urbistondo M., Mellor-Pita S., Moreno-Torres V., de Luis D., Martínez J.A. (2024). Body mass index and fat influences the role of *Bifidobacterium genus* in lupus patients concerning fibrinogen levels. Front. Microbiol..

[34] Chero-Sandoval L., Martínez-Urbistondo M., Cuevas-Sierra A., Higuera-Gómez A., Martin-Domenech E., Castejón R., Mellor-Pita S., Moreno-Torres V., Ramos-Lopez O., de Luis D. (2024). Comparison of Metabolic Syndrome, Autoimmune and Viral Distinctive Inflammatory Related Conditions as Affected by Body Mass Index. J. Clin. Med..

