# Supplementary material for: Polygenic Risk and Linked Metabolic Profile in Systemic Lupus Erythematosus: Cross-Sectional Insights
Source: Genes (Basel). 2026 Jan 1;17(1):53. doi: 10.3390/genes17010053 (PMC12841000; doi:10.3390/genes17010053)
Supplement: Supplementary file 1 [file genes-17-00053-s001.zip › genes-3990505-supplementary.pdf]

**SUPPLEMENTARY MATERIAL:**

**Table S1. Polygenic risk score (PRS) in systemic lupus erythematosus (SLE) and metabolic syndrome (MS) populations**

| | Overall | SLE normal weight | SLE overweight | MetS | P test |
|---|---|---|---|---|---|
| *n* | 93 | 21 | 35 | 37 | |
| **PRS appetite** | 44.0 [19.0, 72.0] | 44.0 [19.0, 72.0] | 44.0 [19.0, 44.0] | 44.0 [19.0, 72.0] | 0.33 |
| **PRS proinflammatory** | 45.0 [31.0, 82.0] | 43.0 [37.0, 57.0] | 74.0 [34.0, 87.5] | 43.0 [12.0, 68.0] | **0.049** |
| **PRS homocysteine** | 51.0 [27.0, 72.0] | 46.0 [26.0, 68.0] | 59.0 [29.5, 75.0] | 49.0 [28.0, 81.0] | 0.81 |
| **PRS SLE** | 88.0 [63.0, 97.0] | 88.0 [73.0, 99.0] | 94.0 [76.5, 98.0] | 73.0 [51.0, 92.0] | **0.018** |
| **PRS glucose** | 51.0 [28.0, 76.0] | 57.0 [35.0, 79.0] | 49.0 [29.0, 74.0] | 56.0 [28.0, 82.0] | 0.61 |
| **PRS insulin** | 43.0 [27.0, 64.0] | 41.0 [22.0, 54.0] | 42.0 [27.5, 58.5] | 53.0 [28.0, 73.0] | 0.45 |
| **PRS CRP** | 53.0 [30.0, 75.0] | 50.0 [28.0, 68.0] | 61.0 [20.5, 77.5] | 53.0 [37.0, 75.0] | 0.64 |
| **PRS ALT** | 52.0 [28.0, 72.0] | 55.0 [41.0, 72.0] | 52.0 [31.5, 67.0] | 45.0 [23.0, 72.0] | 0.41 |
| **PRS AST** | 34.0 [25.0, 60.0] | 40.0 [19.0, 51.0] | 33.0 [17.0, 50.0] | 42.0 [25.0, 74.0] | 0.14 |
| **PRS obesity** | 56.0 [10.0, 56.0] | 56.0 [15.0, 56.0] | 56.0 [10.0, 71.5] | 56.0 [10.0, 56.0] | 0.35 |
| **PRS WHR** | 66.0 [54.0, 85.0] | 62.0 [37.0, 78.0] | 71.0 [55.5, 85.5] | 67.0 [55.0, 88.0] | 0.25 |
| **PRS insulin resistance** | 56.0 [34.0, 77.0] | 46.0 [36.0, 57.0] | 61.0 [21.5, 78.5] | 62.0 [35.0, 78.0] | 0.39 |
| **PRS MS** | 43.0 [21.0, 67.0] | 47.0 [19.0, 70.0] | 34.0 [22.0, 67.5] | 47.0 [19.0, 63.0] | 0.92 |
| **PRS thermogenesis** | 37.0 [37.0, 79.0] | 37.0 [37.0, 79.0] | 37.0 [37.0, 79.0] | 37.0 [37.0, 79.0] | 0.27 |

Raw data. Continuous variables are expressed as median [Q1-Q3], as they were not normally distributed. Comparisons across groups were performed using the Kruskal–Wallis test. Bold numbers mean significant differences. PRS: polygenic risk score. SLE: systemic lupus erythematosus. MS: metabolic syndrome. CRP: C-reactive protein. ALT: alanine aminotransferase. AST: aspartate aminotransferase. WHR: waist-hip ratio.

**Table S2: Systemic lupus erythematosus (SLE) single nucleotide polymorphisms (SNPs) for polygenic risk score (PRS)**

| rsID | Gene | Chr | Position | Risk alelle | Effect size |
|---|---|---|---|---|---|
| **rs1874791** | *IL12RB2* | 1 | 67806432 | A | 0.131028262 |
| **rs6679677** | *PHTF1* | 1 | 114303808 | A | 0.329303747 |
| **rs2476601** | *PTPN22, AP4B1-AS1* | 1 | 114377568 | A | 0.357674444 |
| **rs6671847** | *FCGR2A* | 1 | 161478810 | A | 0.182321557 |
| **rs1801274** | *FCGR2A* | 1 | 161479745 | C | 0.148420005 |
| **rs704840** | *TNFSF4, LOC100506023* | 1 | 173226195 | G | 0.198850859 |
| **rs10912578** | *TNFSF4, LOC100506023* | 1 | 173251856 | A | 0.2390169 |
| **rs10753074** | *TNFSF4, LOC100506023* | 1 | 173346343 | T | 0.19062036 |
| **rs17849501** | *NCF2* | 1 | 183542323 | T | 0.698134722 |
| **rs3024505** | *IL10* | 1 | 206939904 | T | 0.157003749 |
| **rs3024493** | *IL10, IL19* | 1 | 206943968 | A | 0.182321557 |
| **rs4660116** | *LYST* | 1 | 236028841 | C | 0.157003749 |
| **rs9782955** | *LYST* | 1 | 236039877 | C | 0.148420005 |
| **rs7090925** | *LOC105376397* | 10 | 8479868 | A | 0.122217633 |
| **rs7097397** | *WDFY4* | 10 | 50025396 | G | 0.182321557 |
| **rs2663052** | *WDFY4* | 10 | 50069395 | C | 0.148420005 |
| **rs4948496** | *ARID5B* | 10 | 63805617 | C | 0.131028262 |
| **rs10995092** | *RTKN2* | 10 | 64001700 | T | 0.198850859 |
| **rs12802200** | *MIR210HG* | 11 | 566936 | C | 0.207014169 |
| **rs58688157** | *CDHR5* | 11 | 625085 | A | 0.21511138 |
| **rs2732549** | *LOC105376626* | 11 | 35088399 | T | 0.21511138 |
| **rs387619** | *PDHX, CD44* | 11 | 35098193 | C | 0.19062036 |
| **rs494003** | *AP5B1* | 11 | 65542298 | T | 0.122217633 |
| **rs3794060** | *NADSYN1* | 11 | 71187679 | C | 0.207014169 |

| | | | | | |
|---|---|---|---|---|---|
| **rs7941765** | *FLI1, ETS1* | 11 | 128499000 | C | 0.131028262 |
| **rs10774625** | *ATXN2* | 12 | 111910219 | A | 0.122217633 |
| **rs597808** | *ATXN2* | 12 | 111973358 | A | 0.165514438 |
| **rs1059312** | *SLC15A4* | 12 | 129278864 | C | 0.157003749 |
| **rs11059919** | *SLC15A4* | 12 | 129289190 | G | 0.148420005 |
| **rs912784** | *LRRC63, LOC107984578* | 13 | 46785521 | T | 0.254642218 |
| **rs4902562** | *RAD51B* | 14 | 68731458 | A | 0.122217633 |
| **rs2289583** | *SCAMP5* | 15 | 75311036 | A | 0.165514438 |
| **rs9652601** | *CLEC16A* | 16 | 11174365 | G | 0.19062036 |
| **rs7200786** | *CLEC16A* | 16 | 11177801 | A | 0.139761942 |
| **rs34572943** | *ITGAM* | 16 | 31272353 | A | 0.536493371 |
| **rs35472514** | *ITGAM* | 16 | 31283323 | G | 0.530628251 |
| **rs2288012** | *PRSS54* | 16 | 58327646 | A | 0.113328685 |
| **rs13332649** | *IRF8, RPL10AP12* | 16 | 85966683 | A | 0.292669614 |
| **rs11644034** | *IRF8* | 16 | 85972612 | G | 0.223143551 |
| **rs79404002** | *PSMB6, GLTPD2* | 17 | 4697255 | A | 0.254642218 |
| **rs2286672** | *PLD2* | 17 | 4712617 | T | 0.223143551 |
| **rs2941509** | *IKZF3* | 17 | 37921194 | A | 0.300104592 |
| **rs143123127** | *IKZF3* | 17 | 38007190 | A | 0.412109651 |
| **rs959260** | *GRB2* | 17 | 73369422 | A | 0.131028262 |
| **rs11085727** | *TYK2* | 19 | 10466123 | C | 0.21511138 |
| **rs2304256** | *TYK2* | 19 | 10475652 | C | 0.21511138 |
| **rs268134** | *SPRED2* | 2 | 65608363 | G | 0.19062036 |
| **rs2111485** | *LOC105373724* | 2 | 163110536 | G | 0.139761942 |
| **rs1990760** | *IFIH1* | 2 | 163124051 | T | 0.139761942 |
| **rs11889341** | *STAT4* | 2 | 191943742 | T | 0.518793793 |

| | | | | | |
|---|---|---|---|---|---|
| **rs6736175** | *STAT4* | 2 | 191946322 | C | 0.21511138 |
| **rs3768792** | *IKZF2* | 2 | 213871709 | C | 0.21511138 |
| **rs10048743** | *IKZF2* | 2 | 213890232 | G | 0.223143551 |
| **rs11908000** | *PSMB6, GLTPD2* | 20 | 7358423 | T | 1.10856262 |
| **rs7444** | *UBE2L3* | 22 | 21976934 | C | 0.2390169 |
| **rs3747093** | *YDJC* | 22 | 21984379 | A | 0.231111721 |
| **rs9311676** | *LOC107986092* | 3 | 58470351 | C | 0.157003749 |
| **rs77583790** | *IL12A-AS1* | 3 | 159694053 | A | 0.765467842 |
| **rs564799** | *IL12A-AS1* | 3 | 159728987 | C | 0.131028262 |
| **rs10028805** | *BANK1* | 4 | 102737250 | G | 0.182321557 |
| **rs10516487** | *BANK1* | 4 | 102751076 | G | 0.148420005 |
| **rs4388254** | *VDAC1* | 5 | 133428601 | T | 0.336472237 |
| **rs7726414** | *VDAC1* | 5 | 133431834 | T | 0.371563556 |
| **rs6889239** | *TNIP1* | 5 | 150457771 | C | 0.285178942 |
| **rs10036748** | *TNIP1* | 5 | 150458146 | T | 0.322083499 |
| **rs2431697** | *miR-146a, PTTG1* | 5 | 159879978 | T | 0.231111721 |
| **rs1267499** | *LOC101928354* | 6 | 14715882 | A | 0.139761942 |
| **rs74290525** | *SLC44A4* | 6 | 31835162 | G | 0.722705983 |
| **rs1270942** | *CFB* | 6 | 31918860 | C | 0.824175443 |
| **rs1150757** | *TNXB* | 6 | 32029205 | A | 0.845868268 |
| **rs9273076** | *HLA-DQA1* | 6 | 32612301 | T | 0.262364264 |
| **rs9462027** | *UHRF1BP1* | 6 | 34797241 | A | 0.131028262 |
| **rs820077** | *ANKS1A* | 6 | 35033854 | G | 0.173953307 |
| **rs6568431** | *ATG5* | 6 | 106588806 | A | 0.182321557 |
| **rs9398235** | *DDO* | 6 | 110732116 | A | 0.139761942 |
| **rs6914831** | *AHI1* | 6 | 135639644 | C | 0.104360015 |

| | | | | | |
|---|---|---|---|---|---|
| **rs9402743** | *AHI1-DT* | 6 | 136001034 | G | 0.09531018 |
| **rs6932056** | *TNFAIP3* | 6 | 138242437 | C | 0.604315967 |
| **rs58721818** | *TNFAIP3* | 6 | 138243739 | T | 0.598836501 |
| **rs12531540** | *JAZF1* | 7 | 28162674 | C | 0.139761942 |
| **rs849142** | *JAZF1* | 7 | 28185891 | A | 0.131028262 |
| **rs4917014** | *IKZF1* | 7 | 50305863 | T | 0.165514438 |
| **rs11185603** | *IKZF1* | 7 | 50306810 | C | 0.139761942 |
| **rs3757387** | *IRF5* | 7 | 128576086 | C | 0.371563556 |
| **rs35000415** | *IRF5* | 7 | 128585616 | T | 0.604315967 |
| **rs10488631** | *TNPO3* | 7 | 128594183 | C | 0.652325186 |
| **rs2736332** | *BLK, FAM167A* | 8 | 11339965 | C | 0.270027137 |
| **rs2736340** | *BLK, FAM167A* | 8 | 11343973 | T | 0.254642218 |
| **rs2532871** | *BLK, FAM167A* | X | 30573733 | C | 0.277631737 |
| **rs887369** | *TASL* | X | 30577846 | C | 0.139761942 |
| **rs1734787** | *MECP2* | X | 153325446 | C | 0.270027137 |
